# Reduced serum levels of the pro-resolving lipid mediators resolvin D1 and lipoxin A4 in schizophrenia

**DOI:** 10.3389/fpsyt.2026.1943920

**Published:** 2026-10-02

**Authors:** Muhammet Sancaktar, Ece Yildiz, Bahadir Demir, Gulcin Elboga, Feridun Bulbul, Kenan Ercan, Seyithan Taysi, Abdurrahman Altındag

**Affiliations:** 1 Department of Psychiatry, Faculty of Medicine, Gaziantep University, Gaziantep, Türkiye; 2 Department of Biochemistry, Faculty of Medicine, Gaziantep University, Gaziantep, Türkiye

**Keywords:** inflammation, lipoxin A4, resolvin D1, schizophrenia, specialized pro-resolving mediators

## Abstract

Schizophrenia is increasingly conceptualized as a disorder of chronic, unresolved neuroinflammation, yet why the inflammatory burden fails to resolve remains unclear. Specialized pro-resolving lipid mediators—including resolvin D1 (RvD1) and lipoxin A4 (LXA4)—actively terminate inflammation but have rarely been examined in schizophrenia. Because no previous study has measured LXA4 in schizophrenia or assessed both mediators concurrently, we compared serum RvD1 and LXA4 levels between patients and controls and assessed their association with illness severity. In this single-center case-control study, fasting serum RvD1 and LXA4 were measured by ELISA in 35 patients with schizophrenia (DSM-5) during an acute psychotic episode and 35 healthy controls of comparable age and sex; severity was rated with the PANSS and the Clinical Global Impression–Severity (CGI-S) scale. Both mediators were significantly lower in patients than controls (RvD1: 3372 ± 863 vs 3981 ± 382 ng/L, p < 0.001, Cohen’s d = −0.91; LXA4: p = 0.002), and each remained independently associated with case status after adjustment for age, sex, and body mass index. Lower RvD1 correlated with higher CGI-S scores (ρ = −0.54, p = 0.001), whereas LXA4 did not. This provides preliminary evidence that circulating levels of these pro-resolving lipid mediators are reduced in schizophrenia, consistent with altered pro-resolving signalling rather than a demonstrated failure of the resolution process itself. Overall, they identify RvD1 as a candidate severity-related biomarker in patients during an acute psychotic episode, warranting confirmation in larger, longitudinal, mass spectrometry-based studies.

## 1 Introduction

Schizophrenia is an etiologically heterogeneous, chronic disorder characterized by disturbances of perception and thought content and by disorganized behavior, and encompassing genetic, biological, and infectious factors (1). In the central nervous system (CNS), inflammation is carried out in a controlled manner through pro-inflammatory cytokines and immune cells, and many etiological factors such as infection, trauma, or toxins can lead to uncontrolled inflammation (2). In the pathophysiology of schizophrenia, cytokines released from chronically activated macrophages and T-lymphocytes are thought to play a central role by causing abnormalities in neurotransmitter systems (3). Elevated pro-inflammatory cytokine levels in patients with schizophrenia have been demonstrated in studies in the literature, supporting the presence of immune activation and an inflammatory state in the pathophysiology of the disease (4–6).

Lipoxins, resolvins, protectins, and maresins are the subclasses that constitute the specialized pro-resolving mediators (SPMs), lipid derivatives that play a role not only in the resolution of inflammation but also in host defense and tissue repair (7). Reduced SPM levels have been increasingly implicated in neuroinflammatory and neurodegenerative conditions, where they may arise from inadequate substrate availability, an imbalance with pro-inflammatory mediators, or impaired synthesis (8, 9). Notably, biased activation of pro-resolving receptors has recently been proposed as a supportive therapeutic strategy in schizophrenia (10). Docosahexaenoic acid (DHA) and arachidonic acid (AA), which are derivatives of polyunsaturated fatty acids (PUFAs), regulate neuronal activity and gene expression, thereby contributing to the prevention of apoptosis and neuroinflammation and to the modulation of neurogenesis and synaptogenesis (11). It is known that PUFA concentrations in the erythrocyte membranes of patients with schizophrenia are lower than those of healthy individuals (12).

Lipoxin A4 (LXA4) is synthesized from AA, whereas Resolvin D1 (RvD1) is synthesized from DHA, one of the omega-3 fatty acid derivatives (13). LXA4 accelerates the phagocytosis of apoptotic polymorphonuclear leukocytes (PMNs) by macrophages (14), and inhibits neutrophil transmigration and adhesion to the vascular endothelium (15). Similarly, RvD1 limits neutrophil infiltration at inflammatory sites (16). In traumatic brain injury, it protects neurons by preserving the mitochondrial morphology of astrocytes (17). Beyond these effects, RvD1 is regarded as a key endogenous inhibitor of neuroinflammation, with reported plasma changes across mania, depression, and Parkinson’s disease (18).

Although the presence of pro-inflammatory processes in the pathogenesis of schizophrenia is well established, the levels and effects of LXA4 and RvD1, which reflect the resolution capacity of inflammation, have not yet been sufficiently elucidated. Starting from this gap, the aim of our study was to compare serum LXA4 and RvD1 levels in patients with schizophrenia with those of healthy controls and to evaluate the potential role of these molecules in the inflammatory process of the disease and their possible associations with the clinical severity of the illness. To the best of our knowledge, LXA4 has not previously been measured in schizophrenia, and no prior study has assessed RvD1 and LXA4 concurrently in the same cohort; the resolution arm of inflammation therefore remains uncharacterized in this disorder. Because pro-resolving lipid signaling is a conserved biological mechanism rather than a population-specific characteristic, the present findings are expected to be informative for the international schizophrenia research community.

## 2 Methods

### 2.1 Study design

This study was conducted as a single-center, case-control study in the Department of Psychiatry, Sahinbey Research and Training Hospital, Faculty of Medicine, Gaziantep University. All participants were given verbal and written information about the study, and written informed consent was obtained from them (or their legal representatives). The study was carried out in accordance with the principles of the Declaration of Helsinki. The study protocol was approved by the Clinical Research Ethics Committee of Gaziantep University (decision no. 2025/325, dated 10.09.2025).

### 2.2 Sample selection

The sample comprised 35 patients aged 18–65 years, recruited from outpatient and inpatient services, whose diagnosis of schizophrenia was confirmed by two specialist psychiatrists using the SCID-5 (Structured Clinical Interview for DSM-5) and who were in an acute psychotic episode at the time of sampling, together with 35 healthy volunteers selected to be similar to the patients in terms of age and sex distribution. The exclusion criteria for both groups were as follows: (i) any other psychiatric disorder; (ii) intellectual disability; (iii) the presence of acute or chronic infectious, inflammatory, autoimmune, hematological, hepatic, renal, or endocrine disease; (iv) malignancy; (v) cardiovascular disease; (vi) chronic obstructive pulmonary disease; (vii) pregnancy or breastfeeding; (viii) any active substance use disorder other than nicotine; and (ix) use of anti-inflammatory medication.

### 2.3 Clinical assessment

Sociodemographic data of the participants, including age, sex, educational status, body mass index (BMI), and smoking and substance use, as well as clinical data on comorbidity, illness duration, number of psychotic episodes and hospitalizations, and history of ECT, were obtained through face-to-face interviews and file reviews. The severity of clinical symptoms was assessed using the Positive and Negative Syndrome Scale (PANSS) and the Clinical Global Impression-Severity (CGI) scale. All patients were receiving antipsychotic medication at the time of assessment; daily doses were recorded and converted to chlorpromazine (CPZ) equivalents based on published conversion factors (19, 20).

### 2.4 Biochemical measurements

After an overnight fast of at least 8 h, a 5 mL peripheral venous blood sample was drawn from each participant into a serum-separator tube between 08:00 and 10:00. Specimens were left to clot at room temperature for 30 min and then spun at 3,000 × *g* for 10 min; the separated serum was aliquoted and kept at −80 °C until assay. All samples were processed in a single run to minimize between-assay variation. Serum RvD1 and LXA4 were quantified with commercial sandwich ELISA kits (Human Resolvin D1 ELISA Kit, Cat. No. E7450Hu; Human Lipoxin A4 ELISA Kit, Cat. No. E0612Hu; Bioassay Technology Laboratory [BT Lab], Shanghai, China), following the protocol supplied by the manufacturer. Because serum resolvin D1 concentrations exceeded the upper limit of the kit standard curve, samples were diluted five-fold (1:5) with the kit-supplied sample diluent before the resolvin D1 assay, and final concentrations were obtained by multiplying the interpolated values by the dilution factor. Detection ranges and lower sensitivities were 37.5–2400 ng/L (19.01 ng/L) for RvD1 and 5–2000 ng/L (2.47 ng/L) for LXA4. Within- and between-run coefficients of variation stayed below 8% and 10%, respectively. Optical density was read at 450 nm on a BioTek ELx800 microplate reader (BioTek Instruments, Winooski, VT, USA), and concentrations were interpolated from each kit’s standard curve. Both kits are sandwich ELISAs that, according to the manufacturer, are validated for the quantitative detection of the respective analyte in human serum. The manufacturer does not provide quantitative antibody cross-reactivity data against structurally related lipid mediators. All 70 specimens were assayed together in a single run directly from the −80 °C aliquots, so each sample underwent a single freeze–thaw cycle and plate-to-plate variation did not apply. Within the run, patient and control samples were distributed across the plate in a randomized, interleaved layout, and grossly haemolysed specimens were identified visually and excluded before analysis. Because the assay relies on antibody recognition rather than physicochemical separation, and in the absence of manufacturer cross-reactivity data, the ELISA results are best interpreted as supporting relative between-group differences, whereas the absolute concentrations require orthogonal confirmation by liquid chromatography–tandem mass spectrometry (LC-MS/MS).

### 2.5 Statistical analysis

Numerical variables are summarized as mean ± standard deviation or, where distributions were skewed, as median with interquartile range (IQR); categorical variables are given as counts and percentages. Distributional normality of the continuous variables was checked with the Shapiro-Wilk test. Between-group differences were then evaluated with the independent-samples t-test for normally distributed measures—applying the Welch correction when variances were unequal—and with the Mann-Whitney U test when the normality assumption did not hold. Group differences in categorical variables were tested with the chi-square test or, when expected counts were low, Fisher’s exact test. As indices of effect magnitude we report Cohen’s d for parametric and rank-biserial r for non-parametric comparisons. Because the groups differed at baseline in age and body mass index (BMI), the primary biomarker comparisons were re-run in an analysis of covariance (ANCOVA) adjusting for age, sex, and BMI, with partial eta-squared (partial η²) as the effect-size index. Within the patient group, biomarker–clinical relationships were quantified using Spearman’s rank correlation. To limit Type I error across the multiple within-patient correlations, p values were corrected with the Benjamini-Hochberg false discovery rate (FDR) procedure and expressed as q values; coefficients that lost significance after correction are treated as exploratory. The discriminative performance of each biomarker was examined by receiver operating characteristic (ROC) analysis, with the area under the curve (AUC) and its 95% confidence interval derived from 2000 bootstrap resamples and the optimal threshold set by the Youden J index. The independent contribution of each biomarker to case status was modeled with binary logistic regression, both unadjusted and adjusted for age, sex, and BMI. Sample-size adequacy was appraised through a sensitivity analysis in G*Power 3.1 (reported in the Results). Analyses were carried out in SPSS v25.0, and a two-sided p < 0.05 denoted statistical significance.

## 3 Results

### 3.1 Sociodemographic characteristics of the study sample

The study included 35 patients with schizophrenia and 35 healthy controls (total N = 70). The sociodemographic and clinical characteristics of the two groups are presented in **Table 1**. The groups did not differ significantly with respect to sex distribution (χ² = 0.06, p = 0.811), tobacco/alcohol/substance use (χ² = 1.43, p = 0.232), or medical comorbidity (Fisher’s exact p = 1.000). However, patients were significantly older than controls (44.14 ± 10.08 vs. 39.29 ± 5.12 years; Welch t = 2.54, p = 0.014; Cohen’s d = 0.61), and body mass index (BMI) was marginally higher in patients (28.77 ± 5.10 vs. 26.90 ± 3.10 kg/m²; Welch t = 1.86, p = 0.069; Cohen’s d = 0.44). As expected, educational attainment was lower in patients (Mann-Whitney U = 93.0, p < 0.001); given that educational attainment is commonly affected by the disease process itself in schizophrenia, education was not used as a covariate in subsequent adjusted analyses. To address the observed imbalances in age and BMI, group comparisons of the primary biomarker outcomes were repeated within an analysis of covariance (ANCOVA) framework (see Section 3.3). A sensitivity power analysis (G*Power 3.1) indicated that, with 35 participants per group and a two-tailed α of 0.05, the study had 80% power to detect a between-group difference of Cohen’s d ≥ 0.68 and 90% power to detect d ≥ 0.78; for within-group correlations (n = 35), the study had 80% power to detect a coefficient of |ρ| ≥ 0.46. The observed effects for the primary outcomes (RvD1, d = 0.91; RvD1–CGI-S, ρ = −0.539) therefore exceeded these detectable thresholds, whereas smaller associations should be regarded as underpowered.

**Table 1:** Sociodemographic and clinical characteristics of the study groups.

Variable	Patients (n = 35)	Controls (n = 35)	Test	Statistic	p-value
Age (years), mean ± SD	44.14 ± 10.08	39.29 ± 5.12	Welch t	2.541	**0.014**
BMI (kg/m²), mean ± SD	28.77 ± 5.10	26.90 ± 3.10	Welch t	1.856	0.069
Sex (F/M), n	16/19	18/17	Pearson χ²	0.057	0.811
Education level, median (IQR)	3 (2–4)	6 (5–6)	Mann-Whitney U	93.0	**<0.001**
Substance/alcohol/tobacco use (yes/no), n	21/14	15/20	Pearson χ²	1.430	0.232
Medical comorbidity (yes/no), n	5/30	4/31	Fisher’s exact	—	1.000

BMI, body mass index; IQR, interquartile range; SD, standard deviation. Welch’s t-test was used when Levene’s test indicated unequal variances. Bold p-values indicate statistical significance (p < 0.05). Education was coded as an ordinal variable (1 = illiterate; 2 = primary school; 3 = secondary school; 4 = high school; 5 = university; 6 = postgraduate).Bold values indicate statistical significance (p < 0.05).

### 3.2 Clinical characteristics of the patient group

Within the patient group (n = 35), the median illness duration was 18.0 years (interquartile range [IQR]: 8.5–20.0), the median number of lifetime episodes was 5.0 (IQR: 3.5–10.0), and the median number of psychiatric hospitalizations was 2.0 (IQR: 1.0–5.0). The median Clinical Global Impression-Severity (CGI-S) score was 3.0 (IQR: 3.0–6.0), and the mean Positive and Negative Syndrome Scale (PANSS) total score was 74.31 ± 21.25. A total of 21 patients (60.0%) had a history of lifetime electroconvulsive therapy (ECT). Detailed clinical characteristics are presented in **Table 2**.

**Table 2:** Clinical characteristics of the patient group (n = 35).

Variable	Value	Range
Illness duration (years), median (IQR)	18.0 (8.5–20.0)	2.0–40.0
Number of episodes, median (IQR)	5.0 (3.5–10.0)	1.0–30.0
Number of hospitalizations, median (IQR)	2.0 (1.0–5.0)	0.0–20.0
CGI-Severity, median (IQR)	3.0 (3.0–6.0)	2.0–7.0
PANSS total score, mean ± SD	74.31 ± 21.25	42.0–125.0
Lifetime ECT history (yes/no), n	21/14	—

CGI, Clinical Global Impression scale; ECT, electroconvulsive therapy; IQR, interquartile range; PANSS, Positive and Negative Syndrome Scale; SD, standard deviation.

### 3.3 Serum resolvin D1 and lipoxin A4 levels: group comparisons

The Shapiro-Wilk test indicated that serum RvD1 levels were normally distributed in both groups (patients: W = 0.982, p = 0.828; controls: W = 0.968, p = 0.383), whereas serum LXA4 levels deviated from normality in the patient group (W = 0.828, p < 0.001) and were therefore analyzed using the non-parametric Mann-Whitney U test.

Serum RvD1 levels were significantly lower in patients with schizophrenia (3372.30 ± 862.70 ng/L) than in healthy controls (3980.99 ± 382.10 ng/L; Welch t = −3.82, p < 0.001), with a large effect size (Cohen’s d = −0.91). Similarly, serum LXA4 levels were significantly lower in patients (median 293.75 ng/L; IQR: 216.30–444.86) compared with controls (median 531.29 ng/L; IQR: 306.57–736.82; Mann-Whitney U = 345.5, p = 0.002; rank-biserial r = 0.44, indicating a medium-to-large effect). Biomarker distributions across groups are summarized in **Table 3** and illustrated in **Figure 1**.

**Table 3:** Serum resolvin D1 and lipoxin A4 levels in patients versus healthy controls.

Biomarker	Patients (n = 35)	Controls (n = 35)	Test	Statistic	Effect size	p-value
Resolvin D1 (ng/L), mean ± SD	3372.30 ± 862.70	3980.99 ± 382.10	Welch t	−3.82	d = −0.91	**< 0.001**
Lipoxin A4 (ng/L), median (IQR)	293.75 (216.30–444.86)	531.29 (306.57–736.82)	Mann–Whitney U	345.5	r = 0.44	**0.002**

d, Cohen’s d; IQR, interquartile range; r, rank-biserial correlation; SD, standard deviation. Bold p-values indicate statistical significance (p < 0.05).

**Figure 1:**
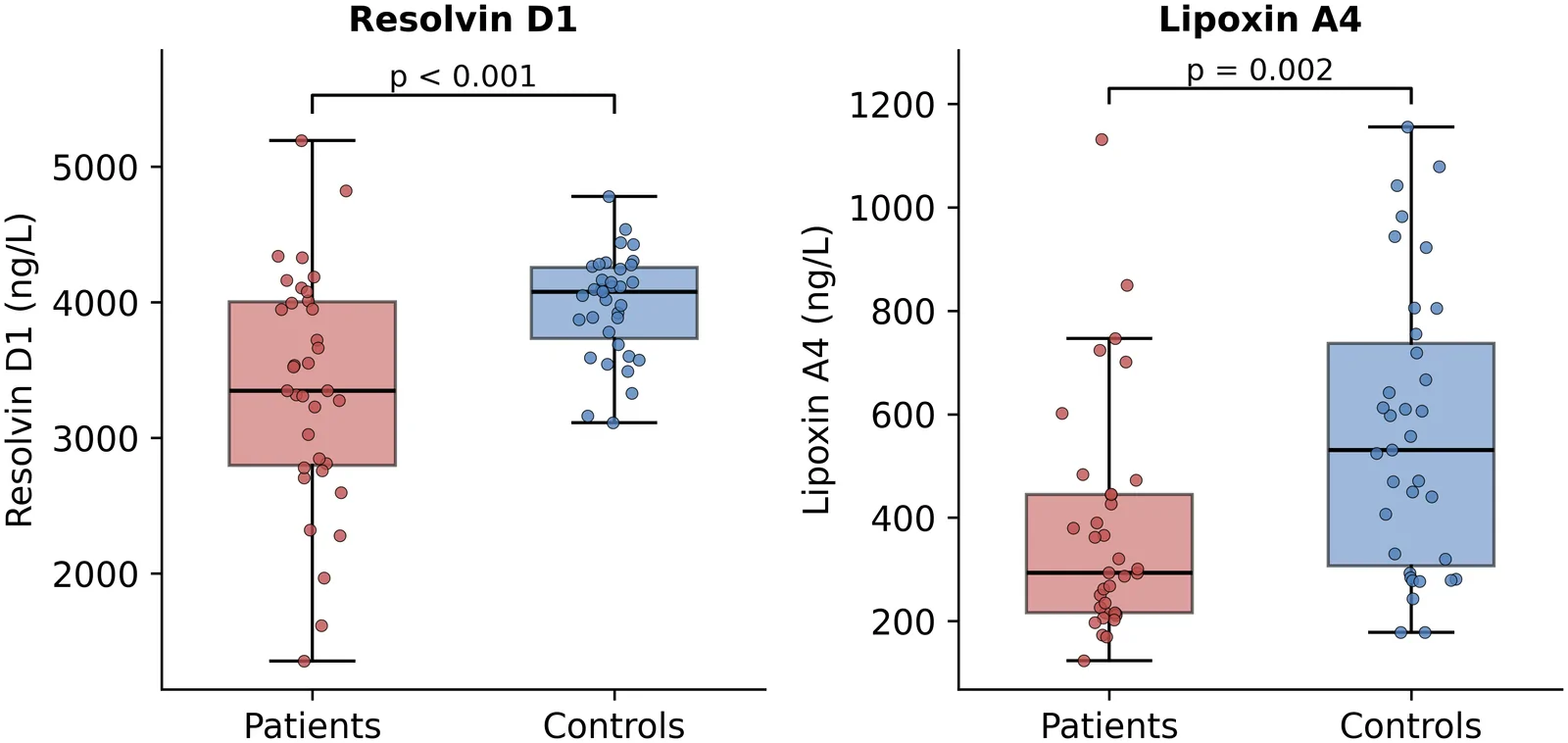
Serum resolvin D1 (left) and lipoxin A4 (right) levels in patients with schizophrenia (n = 35) versus healthy controls (n = 35). Boxes show the median and interquartile range; whiskers show the range; individual values are overlaid. Both mediators were significantly lower in patients (resolvin D1, Welch t-test, p < 0.001; lipoxin A4, Mann–Whitney U test, p = 0.002).

Given the baseline imbalance in age and the marginal difference in BMI between groups (see Section 3.1), an analysis of covariance (ANCOVA) was conducted, adjusting for age, sex, and BMI as covariates (**Table 4**). After covariate adjustment, the effect of group remained highly significant for RvD1 (F(1, 65) = 12.86, p < 0.001, partial η² = 0.165), with adjusted means of 3367.03 ng/L for patients and 3986.27 ng/L for controls. The group effect remained significant for LXA4 as well (F(1, 65) = 8.30, p = 0.005, partial η² = 0.113), with adjusted means of 376.70 ng/L for patients and 564.15 ng/L for controls. None of the covariates (age, sex, BMI) reached statistical significance in either model (all p > 0.05). According to Cohen’s (1988) conventions, the observed partial η² values correspond to a large effect for RvD1 and a medium-to-large effect for LXA4, indicating that the lower biomarker levels observed in patients are not explained by between-group differences in age, sex, or BMI. Because serum LXA4 deviated from normality, the covariate-adjusted group comparison was repeated on log-transformed LXA4 and, additionally, using a rank-based ANCOVA; the group effect remained significant in both (log-transformed: F(1, 65) = 10.73, p = 0.002, partial η² = 0.14; rank-based: p = 0.002), indicating that the lower LXA4 levels in patients are not an artifact of the skewed distribution.

**Table 4:** Analysis of covariance (ANCOVA) for serum Resolvin D1 and Lipoxin A4 levels, adjusting for age, sex, and body mass index.

Outcome	Source	F	df	p-value	Partial η²
Resolvin D1	Group	12.858	1, 65	**<0.001**	0.165
	Age	1.132	1, 65	0.291	0.017
	Sex	0.231	1, 65	0.632	0.004
	BMI	2.582	1, 65	0.113	0.038
Lipoxin A4	Group	8.301	1, 65	**0.005**	0.113
	Age	0.572	1, 65	0.452	0.009
	Sex	0.933	1, 65	0.338	0.014
	BMI	1.659	1, 65	0.202	0.025

BMI, body mass index; df, degrees of freedom. Partial η² interpretation: 0.01 = small, 0.06 = medium, 0.14 = large (Cohen, 1988). Adjusted (estimated marginal) means: Resolvin D1 — Patients 3367.03 ng/L, Controls 3986.27 ng/L; Lipoxin A4 — Patients 376.70 ng/L, Controls 564.15 ng/L. Bold p-values indicate statistical significance (p < 0.05).

### 3.4 Correlations between biomarkers and clinical variables

Within the patient group, the relationships between serum RvD1 and LXA4 levels and demographic and clinical variables were examined using Spearman’s rank correlation as the primary statistic (**Supplementary Table 1**; **Figure 2**). Serum RvD1 levels showed a strong negative correlation with CGI-Severity scores (ρ = −0.539, p = 0.001), indicating that lower RvD1 levels were associated with greater clinician-rated illness severity. RvD1 levels showed non-significant trends toward a negative correlation with the number of lifetime episodes (ρ = −0.302, p = 0.078) and a positive correlation with BMI (ρ = +0.318, p = 0.063). RvD1 was not significantly correlated with age, illness duration, number of hospitalizations, or PANSS total score (all p > 0.10).

**Figure 2:**
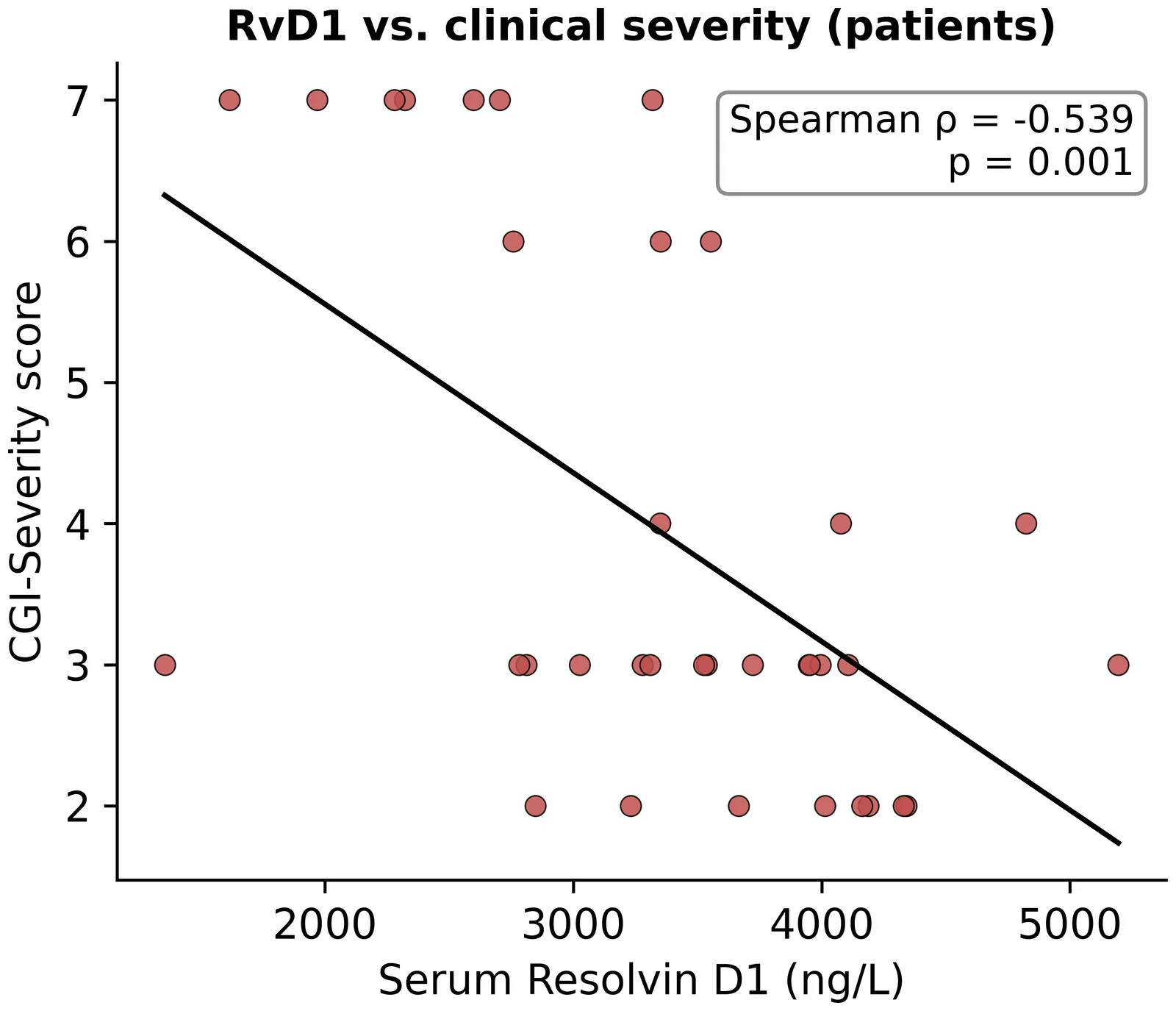
Scatterplot of serum resolvin D1 against the Clinical Global Impression–Severity (CGI-S) score within the patient group (n = 35). Lower resolvin D1 levels were associated with greater illness severity (Spearman ρ = −0.539, p = 0.001); the solid line indicates the linear fit.

Serum LXA4 levels showed a significant positive correlation with BMI (ρ = +0.377, p = 0.026) but were not significantly correlated with age, illness duration, number of episodes, number of hospitalizations, CGI-Severity, or PANSS total score (all p > 0.10). The two biomarkers were not significantly correlated with each other within the patient group (ρ = +0.034, p = 0.848), suggesting that they may capture partially independent aspects of the specialized pro-resolving mediator (SPM) pathway. After Benjamini-Hochberg correction for multiple comparisons within the patient group, only the negative association between RvD1 and CGI-Severity remained statistically significant (q < 0.05); the positive LXA4–BMI correlation did not survive correction and is therefore interpreted as an exploratory finding requiring replication. Within the patient group, the median chlorpromazine-equivalent antipsychotic dose was 965 mg/day (interquartile range 450–1600; range 50–2700). Neither serum RvD1 (ρ = −0.213, p = 0.219) nor LXA4 (ρ = +0.257, p = 0.135) was significantly correlated with the chlorpromazine-equivalent dose, whereas the dose was, as expected, positively associated with symptom severity (CGI-Severity, ρ = +0.362, p = 0.033; PANSS total score, ρ = +0.471, p = 0.004). Moreover, the inverse association between RvD1 and CGI-Severity remained significant after controlling for the chlorpromazine-equivalent dose (partial ρ = −0.508, p = 0.002), indicating that it was independent of antipsychotic dose. With respect to antipsychotic class, 31 patients (88.6%) were receiving second-generation (atypical) antipsychotics only, whereas 4 (11.4%) were additionally exposed to a first-generation agent (haloperidol); 17 (48.6%) were receiving clozapine and 29 (82.9%) were on antipsychotic polypharmacy. In exploratory subgroup comparisons, serum RvD1 did not differ between clozapine users and non-users (medians 3536 vs 3350 ng/L, p = 0.86) or between patients with versus without first-generation exposure (p = 0.71), and serum LXA4 did not differ by clozapine use (p = 0.59); the nominally higher LXA4 in the four patients with first-generation exposure (p = 0.049) rests on a very small subgroup and is not interpretable. Because almost all patients were receiving atypical antipsychotics, a formal typical-versus-atypical comparison was not feasible, and these stratified analyses are underpowered and reported only as sensitivity checks.

### 3.5 Diagnostic performance: receiver operating characteristic analysis

To evaluate the ability of each biomarker to discriminate patients with schizophrenia from healthy controls, receiver operating characteristic (ROC) curves were generated (**Supplementary Table 2**; **Figure 3**). Serum RvD1 yielded an area under the curve (AUC) of 0.739 (95% CI: 0.613–0.852; Hanley-McNeil p < 0.001), with an optimal cutoff of ≤3536.29 ng/L (Youden’s J = 0.457) yielding a specificity of 88.6% and a sensitivity of 57.1%. Serum LXA4 yielded an AUC of 0.718 (95% CI: 0.594–0.830; Hanley-McNeil p < 0.001), with an optimal cutoff of ≤445.37 ng/L (Youden’s J = 0.400) yielding a sensitivity of 77.1% and a specificity of 62.9%. According to conventional classification (Hosmer & Lemeshow, 2013), both biomarkers showed only modest-to-acceptable discriminative ability (AUC = 0.70–0.80), with wide confidence intervals and limited sensitivity (RvD1, 57.1%). These figures indicate group-level differences rather than stand-alone diagnostic utility; the values are not sufficient for individual-level classification and are reported here as exploratory indicators of discriminative potential that would require validation in larger, independent samples before any clinical application. Because the AUC values, optimal thresholds, and their sensitivity and specificity were all derived from the same single dataset, they are internally derived, optimistic estimates; the reported cutoffs are not externally validated clinical thresholds and require confirmation in independent samples.

**Figure 3:**
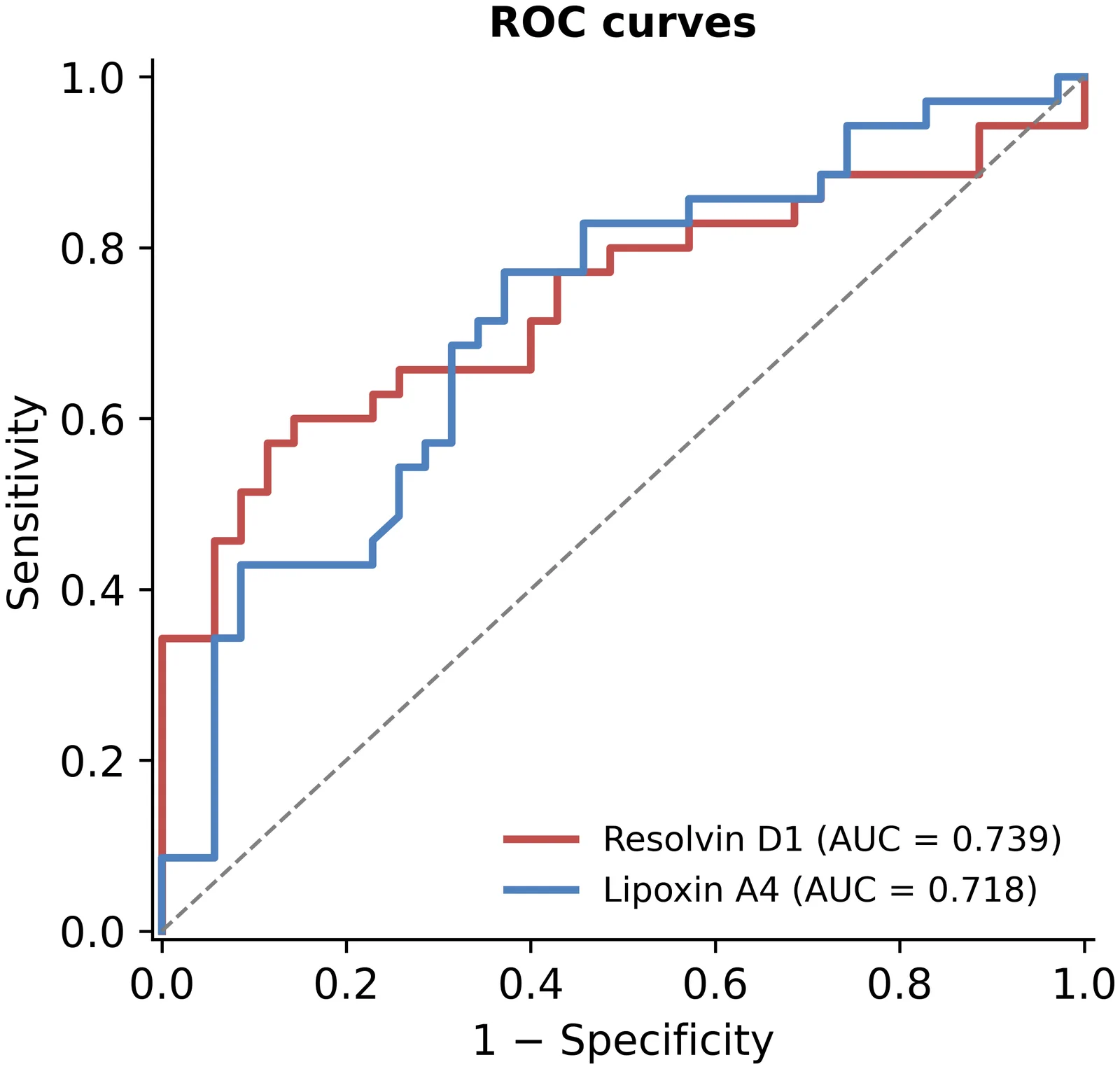
Receiver operating characteristic (ROC) curves of serum resolvin D1 and lipoxin A4 for discriminating patients with schizophrenia from healthy controls. Areas under the curve were 0.739 and 0.718, respectively.

### 3.6 Independent association of biomarkers with case status

To assess the independent association of each biomarker with patient (case) status, univariate and multivariate binary logistic regression models were fitted (**Table 5**). In univariate models, both biomarkers were significantly associated with patient status: for each 100 ng/L increase, the odds of being a patient decreased by 14.3% for RvD1 (OR = 0.857; 95% CI: 0.779–0.944; p = 0.002) and by 26.6% for LXA4 (OR = 0.734; 95% CI: 0.591–0.913; p = 0.005). After adjustment for age, sex, and BMI, both biomarkers retained their independent association with case status. In the RvD1-adjusted model, each 100 ng/L increase in RvD1 was independently associated with lower odds of being a patient (OR = 0.851; 95% CI: 0.768–0.943; p = 0.004). In the LXA4-adjusted model, each 100 ng/L increase in LXA4 was independently associated with lower odds of being a patient (OR = 0.724; 95% CI: 0.564–0.928; p = 0.010). Older age (OR = 1.09 per year; p = 0.035 and p = 0.022 in the two models) and higher BMI (OR = 1.21 per kg/m²; p = 0.008 in both models) were also independently associated with patient status, consistent with the baseline imbalances. These findings further support the conclusion that the between-group differences in serum RvD1 and LXA4 are not attributable to confounding by age, sex, or BMI. RvD1 and LXA4 were entered in separate adjusted models rather than simultaneously; accordingly, these results indicate that each biomarker is statistically associated with case status after adjustment for age, sex, and BMI, and should not be interpreted as evidence of an independent mechanistic effect.

**Table 5:** Univariate and adjusted logistic regression analyses for biomarker association with patient status.

Model	Variable	B (SE)	OR per 100 ng/L (95% CI)	OR per 1 unit	p-value
Univariate	RvD1	−0.0015 (0.0005)	0.857 (0.779–0.944)	0.998	**0.002**
Univariate	LXA4	−0.0031 (0.0011)	0.734 (0.591–0.913)	0.997	**0.005**
Adjusted (RvD1 + age + sex + BMI)	Age (years)	+0.087 (0.041)	—	1.090	**0.035**
	Sex	+0.580 (0.604)	—	1.786	0.336
	BMI	+0.188 (0.071)	—	1.207	**0.008**
	RvD1	−0.0016 (0.0006)	0.851 (0.768–0.943)	0.998	**0.004**
Adjusted (LXA4 + age + sex + BMI)	Age (years)	+0.086 (0.037)	—	1.090	**0.022**
	Sex	+0.133 (0.572)	—	1.142	0.816
	BMI	+0.190 (0.072)	—	1.209	**0.008**
	LXA4	−0.0032 (0.0013)	0.724 (0.564–0.928)	0.997	**0.010**

B, regression coefficient; BMI, body mass index; CI, confidence interval; LXA4, Lipoxin A4; OR, odds ratio; RvD1, Resolvin D1; SE, standard error. Sex coded as 1 = female, 2 = male. OR per 100 ng/L was calculated as exp(100 × B) to facilitate clinical interpretation. Bold p-values indicate statistical significance (p < 0.05).Bold values indicate statistical significance (p < 0.05).

## 4 Discussion

The main finding of this study is that patients with schizophrenia show reduced serum RvD1 and LXA4 levels; this supports the possibility that a ‘defective resolution’ of inflammation may be involved in the pathophysiology of the disease, although a cross-sectional design cannot demonstrate impairment of the resolution process itself. The reduction in these mediators was not merely a statistical difference; this was further supported by the fact that, in the logistic regression analysis, both mediators remained associated with case status independently of age, sex, and BMI. Although this independent association indicates that low pro-resolving mediator levels co-occur robustly and consistently with schizophrenia, it should be emphasized that, owing to the cross-sectional nature of the study, no causal direction can be attributed to this relationship; that is, it remains unclear whether the low mediator levels predispose to the disease or are a consequence of the disease process. These findings, obtained during an acute psychotic episode, are consistent with reduced circulating levels of mediators that contribute to the resolution of inflammation in schizophrenia and suggest that not only pro-inflammatory activation but also a possible reduction in pro-resolving capacity may be relevant to the pathophysiology of the disease. Notably, neither pro-resolving mediator was correlated with the chlorpromazine-equivalent antipsychotic dose, and the inverse RvD1–severity association persisted after adjustment for dose; these findings argue against antipsychotic exposure being the principal driver of the reduced mediator levels, although, because all patients were medicated, a contribution of medication cannot be entirely excluded. Several studies in the literature have examined RvD1 and LXA4 levels in various psychiatric and neurological diseases. In a study measuring RvD1 levels in bipolar patients during the manic, depressive, and euthymic phases, RvD1 levels were found to be increased relative to healthy controls during the manic and depressive phases, whereas no significant difference was observed during the euthymic phase. This was interpreted to suggest that RvD1 acts as a delayed resolver during acute episodes (21).

In a study conducted in patients with multiple sclerosis, the cerebrospinal fluid (CSF) RvD1 level was significantly higher in the highly active patient group than in the low-activity group, whereas no significant difference was found in LXA4 levels (22). It is noteworthy that RvD1 levels were found to be low even though our patients were in an acute psychotic episode, given that in the bipolar disorder and multiple sclerosis studies summarized above, RvD1 levels tend to rise in a compensatory manner during acute or highly active periods. This directional difference may arise from a failure of the pro-resolving capacity to mount a compensatory response to acute exacerbation in schizophrenia; that is, from a relatively stable (trait-like) resolution deficit rather than from momentary fluctuations. However, this interpretation is speculative; the heterogeneity of these studies with respect to disease, sampled tissue (peripheral blood or CSF), and clinical phase limits direct comparison. Future studies using CSF samples and longitudinal designs are needed to confirm the extent to which the peripheral serum findings obtained in our study reflect central neuroinflammation and resolution dynamics. Consistent with this pattern, a recent study reported that serum RvD1 was significantly elevated in adolescents with first-episode, medication-naïve major depressive disorder and decreased after fluoxetine treatment, indicating that RvD1 may rise reactively during acute affective episodes and is modulated by pharmacological treatment (23).

"A further consideration concerns the relationship between peripheral and central pro-resolving mediator levels. Our measurements were obtained from serum, and it cannot be assumed that circulating RvD1 and LXA4 concentrations directly mirror those in the central nervous system. SPMs and their fatty-acid precursors can cross the blood–brain barrier, and SPMs are also synthesized locally by microglia and astrocytes, so peripheral levels may reflect systemic lipid metabolism, central processes, or both (8, 9). The present peripheral reductions are therefore best interpreted as a systemic signature that is hypothesized—but not demonstrated here—to be linked to central neuroimmune processes; clarifying the correspondence between serum and cerebrospinal fluid or brain SPM levels will require paired central–peripheral sampling.

In an *in vivo* study conducted in rats, RvD1 was found to increase the level of its target receptor, the lipoxin A4/formyl peptide receptor 2 (FPR2/ALX), in remote regions of the brain, and the *in vivo* administration of RvD1 was found to support functional recovery and neuroprotection by reducing microglial activation and preventing inflammation-induced neuronal cell death in remote regions (24). Because RvD1 was measured in serum, any link to central ALX/FPR2 signalling remains indirect; nevertheless, lower RvD1 levels may be consistent with reduced activation of this axis and, potentially, with microglial activation, which could in turn relate to clinical status. In line with this possibility, lower RvD1 levels were significantly correlated with higher CGI-S scores. Although this association suggests that RvD1 may be a candidate marker related to disease severity in schizophrenia, because the cross-sectional design does not permit causal inference and the sample was relatively small, this inference needs to be confirmed by longitudinal studies with larger samples.

Alzheimer’s disease (AD) is the most common type of dementia and is accompanied by inflammation with increased microglial activation in the brain. In a study of patients with AD mild cognitive impairment (MCI) and subjective cognitive impairment (SCI), the LXA4 level in the cerebrospinal fluid (CSF) was found to be lower in the dementia group than in the non-dementia group (MCI and SCI) and no significant difference was observed in RvD1 levels. However, individual differences were much more pronounced in the MCI and SCI groups compared to the AD group. Nevertheless, a significant positive correlation with Mini-Mental State Examination scores was found for both molecules (25).

To the best of our knowledge, this study is the first to evaluate both RvD1 and LXA4—pro-resolving lipid mediators that actively terminate inflammation—together in patients with schizophrenia; in addition, LXA4 was measured for the first time in the context of schizophrenia. Our findings in schizophrenia present a distinct but related profile: although both serum LXA4 and RvD1 levels were significantly decreased, only RvD1 showed a significant correlation with CGI-S. This specific correlation suggests that, although the overall resolution capacity is weakened in schizophrenia, the DHA-derived pathway (RvD1) may be more closely related to overall symptom severity than the AA-derived pathway (LXA4). A finding that warrants explanation here is that RvD1 was significantly correlated with CGI-S but showed no significant association with the PANSS total score. This apparent inconsistency may be interpreted in several ways: CGI-S reflects the clinician’s holistic (global) assessment of the patient’s overall illness burden and functional impairment, whereas the PANSS total score combines the positive, negative, and general psychopathology dimensions—which may be associated to differing degrees with inflammatory processes—into a single composite score, which may dilute a possible association. Therefore, our findings suggest that RvD1 may be related to overall clinical severity and illness burden rather than to a specific symptom dimension. However, because this distinction is based on a single global scale in a relatively small sample, it should be interpreted with caution and confirmed in independent samples using dimensional scales. In addition, with only 35 patients the analysis was underpowered to detect weaker correlations, so the absence of a significant RvD1–PANSS association may reflect limited statistical power rather than a true absence of association; both possibilities should be considered.

Normal neuronal phospholipid metabolism is essential for neuromodulation and neuronal function. The role of lipid abnormalities in the pathophysiology of schizophrenia was first emphasized by Horrobin’s ‘Membrane Phospholipid Hypothesis,’ according to which there is an increased rate of loss of AA and DHA in neurons in schizophrenia (26). In line with this, a recent study in patients with schizophrenia reported decreased n-3 PUFA content and reduced membrane fluidity alongside a pro-inflammatory cytokine profile (27).

As noted above, AA and DHA serve as precursors for the synthesis of the specialized lipid mediators (LXA4 and RvD1) that actively terminate inflammation (7). In our study, the finding that both mediators were systemically reduced in patients with schizophrenia may be regarded as a direct reflection of this membrane phospholipid depletion. On the other hand, the positive correlation we found between LXA4 levels and BMI may result from increased adipose tissue providing a larger reservoir of the lipid precursors required for LXA4 synthesis. A study in mice showed that dietary deprivation of PUFAs (AA and DHA) during the gestational and early postnatal periods produced behavioral signs reminiscent of early psychosis in humans (the prodromal phase of schizophrenia) and impairments in cognitive processing by affecting the epigenetic regulation of nuclear receptor genes (28). DHA is the principal omega-3 polyunsaturated fatty acid found in the gray matter of mammals. In first-episode schizophrenia patients, the addition of omega-3 to treatment has been shown to improve PANSS, CGI, and depressive symptoms (29). A meta-analysis of 20 randomized controlled trials (1,494 patients) likewise found that omega-3 supplementation improves general psychopathology and positive symptoms in schizophrenia (30). In patients at high risk of psychotic disorder, omega-3 supplementation significantly reduced the risk of progression to illness and provided symptomatic and functional improvement (31). However, in the NEURAPRO trial the anti-inflammatory effect of omega-3 fatty acids on plasma immune markers in clinical high-risk individuals did not translate into clinical benefit, underscoring the complexity of this relationship (32). In a more recent study, although the levels of long-chain omega-3 and omega-6 fatty acids were found to be associated with a lower risk of schizophrenia, the omega-3 fatty acid DHA was observed to have a more protective effect (33). In light of our finding that only DHA-derived RvD1 showed a significant negative correlation with CGI-S scores, it is possible that the therapeutic benefits observed in omega-3 supplementation studies are partly mediated by a substrate-driven, obligatory increase in RvD1 synthesis. Supporting a mediator-based mechanism, EPA and DHA exert anti-inflammatory and neurogenic effects in human hippocampal cells and in patients with depression through their LOX- and CYP450-derived lipid metabolites (34).

It should also be emphasized that, because RvD1 and LXA4 are synthesized from DHA and AA, respectively, the lower mediator levels we observed could partly reflect reduced dietary intake or availability of these precursor fatty acids rather than a specific failure of the resolution machinery. As dietary fatty-acid intake was not recorded, precursor-driven and resolution-driven explanations cannot be distinguished in the present data; this also tempers the interpretation of the omega-3 supplementation findings discussed above.

### 4.1 Limitations

Our study has some methodological limitations. First, the single-center nature of the study and its relatively small sample size (35 patients and 35 healthy controls) limit the generalizability of the findings. Second, owing to the cross-sectional case-control design, a causal relationship between the reduction in serum RvD1 and LXA4 levels and disease severity cannot be definitively established. Third, although the synthesis of LXA4 and RvD1 directly depends on dietary PUFA intake, the participants’ dietary habits and daily fatty acid intake were not recorded. Fourth, although antipsychotic doses were recorded and expressed as chlorpromazine equivalents—and were not correlated with serum RvD1 or LXA4 levels—all patients were receiving antipsychotic treatment and no drug-naïve comparison group was available; a medication-class effect on the measured mediators therefore cannot be entirely excluded. In addition, although the groups did not differ in combined tobacco, alcohol, and substance use, smoking status was not recorded separately and pack-years were unavailable; given the effects of nicotine on lipid and inflammatory pathways, residual confounding by smoking cannot be excluded. Finally, because the sample consisted only of patients in an acute psychotic episode, the generalizability of the findings to the remission period and to other stages of the disease is limited. A further limitation concerns measurement: serum RvD1 and LXA4 were quantified by ELISA rather than by liquid chromatography-tandem mass spectrometry (LC-MS/MS), which is regarded as the reference method. Given the very low circulating concentrations and instability of SPMs and the ongoing debate regarding their reliable quantification and biology, our absolute values should be interpreted with caution and confirmed using mass spectrometry-based lipidomics (35).

## 5 Conclusion

In conclusion, our study demonstrates that the inflammation-resolving mediators LXA4 and RvD1 are reduced in the serum of patients with schizophrenia. However, the clinically most notable finding of this study is that, although both lipid pathways are reduced, CGI-S scores correlated negatively only with DHA-derived RvD1. This specific association suggests that the DHA-derived omega-3/RvD1 pathway may contribute to clinical severity in schizophrenia, although this cross-sectional finding cannot establish it as the principal mechanism determining clinical course. Furthermore, the reduction in serum LXA4 levels indicates that it is not limited to RvD1 but may extend to a broader network of pro-resolving mediators. In this context, it would be valuable to further investigate these pro-resolving mediators, particularly RvD1, as candidate biomarkers for monitoring disease severity and assessing treatment response; however, the present findings are not sufficient for individual diagnostic use and need to be validated in larger, independent samples. Prospective studies that also include CSF measurements are needed to fully elucidate the causality of these peripheral findings and their dynamics within the central nervous system.

## Data Availability

The raw data supporting the conclusions of this article will be made available by the authors, without undue reservation.
