# Supplementary material for: Reduced serum levels of the pro-resolving lipid mediators resolvin D1 and lipoxin A4 in schizophrenia
Source: Front Psychiatry. 2026 Oct 2;17:1943920. doi: 10.3389/fpsyt.2026.1943920 (PMC13674342; doi:10.3389/fpsyt.2026.1943920)
Supplement: Supplementary file 1 [file Table1.docx]

**Supplementary Material (Online Resources)**

**Table S1 (Online Resource 1). Spearman correlations between serum biomarkers and clinical variables within the patient group (n = 35).**

| **Variable** | **Biomarker** | **N** | **ρ** | **95% CI*** | **p-value** |
| --- | --- | --- | --- | --- | --- |
| Age (years) | RvD1 | 35 | −0.217 | (−0.51, +0.13) | 0.210 |
|  | LXA4 | 35 | −0.175 | (−0.48, +0.17) | 0.314 |
| BMI (kg/m²) | RvD1 | 35 | +0.318 | (−0.02, +0.59) | 0.063 |
|  | LXA4 | 35 | +0.377 | (+0.05, +0.63) | **0.026** |
| Illness duration (years) | RvD1 | 35 | −0.125 | (−0.44, +0.22) | 0.476 |
|  | LXA4 | 35 | −0.255 | (−0.54, +0.09) | 0.140 |
| Number of episodes | RvD1 | 35 | −0.302 | (−0.57, +0.04) | 0.078 |
|  | LXA4 | 35 | +0.044 | (−0.29, +0.37) | 0.801 |
| Number of hospitalizations | RvD1 | 35 | −0.226 | (−0.52, +0.12) | 0.192 |
|  | LXA4 | 35 | +0.232 | (−0.11, +0.52) | 0.179 |
| **CGI-Severity** | RvD1 | 35 | −0.539 | (−0.74, −0.24) | **0.001** |
|  | LXA4 | 35 | +0.082 | (−0.26, +0.40) | 0.639 |
| PANSS total score | RvD1 | 35 | −0.161 | (−0.47, +0.18) | 0.355 |
|  | LXA4 | 35 | +0.159 | (−0.19, +0.47) | 0.361 |

Note. BMI, body mass index; CGI, Clinical Global Impression; LXA4, Lipoxin A4; PANSS, Positive and Negative Syndrome Scale; RvD1, Resolvin D1. *Approximate 95% confidence intervals derived using Fisher's z-transformation. Bold values indicate statistical significance (p < 0.05).

**Table S2 (Online Resource 2). Diagnostic performance of serum Resolvin D1 and Lipoxin A4 in discriminating patients with schizophrenia from healthy controls.**

| **Biomarker** | **AUC** | **95% CI** | **Cutoff (ng/L)** | **Sensitivity** | **Specificity** | **p-value** |
| --- | --- | --- | --- | --- | --- | --- |
| Resolvin D1 | 0.739 | 0.613–0.852 | ≤ 3536.29 | 57.1% | 88.6% | **<0.001** |
| Lipoxin A4 | 0.718 | 0.594–0.830 | ≤ 445.37 | 77.1% | 62.9% | **<0.001** |

Note. AUC, area under the receiver operating characteristic curve; CI, confidence interval. Optimal cutoff values were determined using Youden's J statistic (J = sensitivity + specificity − 1). 95% CIs for AUC were estimated using 2000-iteration non-parametric bootstrap resampling; p-values were calculated using the Hanley-McNeil method. Bold p-values indicate statistical significance (p < 0.05).
